# Miscellany

**Published:** 1892-06

**Authors:** 


### Miscellany.

Messrs. D. Appleton & Co. announce the following in press: A Treatise on Diseases of the Rectum, Anus, and Sigmoid Flexure, by Joseph M. Mathews, M. D., of Louisville, Ky., Professor of the Principles and Practice of Surgery, and Clinical Lecturer on Diseases of the Rectum, in the Kentucky School of Medicine, etc. The work will be illustrated by six chromo-lithographs, showing operations on fistula, hemorrhoids, and other affections of the rectum, and by numerous cuts in the text. Dr. Mathews has devoted many years to the study and teaching of the diseases which are the subject of the treatise, and has written a work that is unique in its excellence and fully up to date. His prominence in the medical profession as a surgeon, teacher, and specialist, insures the success of the work. It is expected that the book will be ready during the present month.

Messrs. P. Blakiston, Son & Co. announce the following important new text-book: Materia Medica, Pharmacy, Pharmacology, and Therapeutics. By Wm. Hale White, M. D., F. R. C. P., etc., Physician to, and Lecturer on Materia Medica at, Guy's Hospital; Examiner in Materia Medica, Royal College of Physicians and Royal College of Surgeons, etc. American Copyright Edition, edited by Reynold W. Wilcox, M. A., M. D., Professor of Clinical Medicine at the New York Post-Graduate Medical School and Hospital, Assistant Visiting Physician, Bellevue Hospital, etc. To be printed in one compact, handy volume. They also announce that a German edition of the second revision of Gower's Book on the Nervous System has just been published by Cohen, of Bonn, (Vol. I. has already appeared,) and we understand that an Italian translation is nearly ready.

---

The April (1892) number of *The Alienist and Neurologist* contains: Surgical Cure of Mental Maladies—Résumé, by Dr. Guiseppe Seppilli, Italy; Some Principles Involved in the Nature and Treatment of Inebriety, by T. L. Wright, M. D., Bellefontaine, Ohio; Art in the Insane, by J. G Kiernan, M. D., Chicago, Ill.; Drug Habituation, by Lucius W. Baker, M. D., Baldwinville, Mass.; Tumor of the Cerebellum, by George J. Preston, M. D., Baltimore; The Epidemic Inflammatory Neurosis, or Neurotic Influenza, by C. H. Hughes, M. D., St. Louis; Pessimism in its Relation to Suicide, by Wm. W. Ireland, M. D., Scotland; Classification of Insanity, by C. G. Chaddock, Traverse City, Mich.; Report of a Case of Transitory Frenzy, by Theo. Diller, M. D., Pittsburgh; Intermittent Paralysis, by L. Bremer, M. D., St. Louis. Besides the usual selections, editorials, hospital notes, reviews, etc.

---

The Dios Chemical Co. publishes a chart of cerebral localization in colors, according to the authority of Horsley, Beever, and Schafer. This chart will be furnished free on application to the publishers, 914 Locust street, St. Louis, Mo.

---

The J. B. Lippincott Company, of Philadelphia, announce that Vol. II., Regional Anatomy, by Dr. Geo. McClellan, is now ready for distribution to the subscribers to that admirable work. As it is only sold on subscription, prompt orders are desirable.

---

It will be of great interest to the profession to learn that Southern Pines, N. C., the new health resort for consumptives, is so located as to be the best known spot during the summer months for those suffering from throat and pulmonary diseases, hay fever and similar maladies. The place is said to be entirely free from malarial influences, and during summer weather patients experience a greater benefit than at any other season, probably due to the fact that the warmth of summer causes the atmosphere to be loaded with the balsamic qualities of the pines. The testimony of physicians of experience would seem to warrant a trial of this resort in cases where a change of climate is desirable. The thermometer rarely goes above ninety degrees in the shade.

